# Prevalence and risk factors of intestinal protozoan infection among symptomatic and asymptomatic populations in rural and urban areas of southern Algeria

**DOI:** 10.1186/s12879-021-06615-5

**Published:** 2021-08-30

**Authors:** Soumia Sebaa, Jerzy M. Behnke, Djamel Baroudi, Ahcene Hakem, Marawan A. Abu-Madi

**Affiliations:** 1 Laboratory of Exploration and Valorization of Steppic Ecosystems, Faculty SNV, University of Ziane Achour, 17000 Djelfa, Algeria; 2 School of Life Sciences, University of Nottingham, University Park, Nottingham, NG7 2RD UK; 3 Food Hygiene Laboratory Quality Analysis System, National Veterinary School, Algiers, Algeria; 4 Research Center in Agropastoralism, Moudjbara Road, 17000 Djelfa, Algeria; 5 Department of Biomedical Science, College of Health Sciences, Biomedical and Pharmaceutical Research Unit, QU-Health, Qatar University, P.O. Box 2713, Doha, Qatar

**Keywords:** Intestinal parasites, Symptomatic, Asymptomatic, Animal contact, Rural areas, Temporal changes, Algeria

## Abstract

**Background:**

Intestinal parasitic infections are amongst the most common infections worldwide and have been identified as one of the most significant causes of morbidity and mortality among disadvantaged populations. This comparative cross-sectional study was conducted to assess the prevalence of intestinal protozoan infections and to identify the significant risk factors associated with intestinal parasitic infections in Laghouat province, Southern Algeria.

**Methods:**

A comparative cross-sectional study was conducted, involving 623 symptomatic and 1654 asymptomatic subjects. Structured questionnaires were used to identify environmental, socio demographic and behavioral factors. Stool specimens were collected and examined using direct wet mount, formalin-ether concentration, xenic in vitro culture and staining methods.

**Results:**

A highly significant difference of prevalence was found between symptomatic (82.3%) and asymptomatic subjects (14.9%), with the majority attributable to protozoan infection. The most common species in the symptomatic subjects were *Blastocystis* spp. (43.8%), *E. histolytica/dispar* (25.4%) and *Giardia intestinalis* (14.6%) and more rarely *Enterobius vermicularis* (02.1%), *Teania* spp. (0.6%) and *Trichuris trichiura* (0.2%), while in asymptomatic population *Blastocystis* spp. (8%), *Entamoeba coli* (3.3%) and *Entamoeba histolytica/dispar* (2.5%) were the most common parasites detected with no case of helminth infection. Multivariate log-linear analysis showed that contact with animals was the main risk factor for transmission of these protozoa in both populations. Furthermore, living in rural areas was significantly associated with combined protozoan infection in the asymptomatic population, whereas, in the symptomatic population an increasing trend of protozoan infection was detected in the hot season. In addition, *Blastocystis* spp. and *G. intestinalis* infection were found to be associated with host sex and contact with animals across the study period.

**Conclusions:**

Based on these results, several strategies are recommended in order to effectively reduce these infections including good animal husbandry practices, health education focused on good personal hygiene practices and adequate sanitation.

## Background

Intestinal parasitic infections are amongst the most common infections worldwide causing significant morbidity and mortality [1, 2]. Intestinal parasites cause serious public health problems ranging from diarrhea to impaired cognitive development, iron deficiency anemia and other physical and mental health problems [3]. Common intestinal parasites with direct life cycles, are transmitted mostly by the fecal oral route through direct contact with infected persons or animals, or indirectly through ingestion of contaminated water or food. *Giardia intestinalis* is a frequent cause of diarrhea, affecting approximately 200 million people worldwide [4]. *Blastocystis* spp. has been described as one of the most common eukaryotic organism in human fecal samples and a wide range of animals, although pathogenicity attributable to infection with *Blastocystis* has been the subject of debate [5]. Among parasitic diseases, deaths attributable to intestinal amebiasis, caused by *Entamoeba histolytica*, are only exceeded by those from malaria and schistosomiasis [6]. Approximately 40 million people, worldwide suffer annually from infection with this species and 40,000 die due to the resulting dysentery and liver abscesses [7].

Generally, the distribution and prevalence of various species of intestinal parasites differ from country to country and even regionally within countries because of several environmental, social, and geographical factors [8]. However, many studies in different parts of the world have shown that age [9], host sex [10, 11], poor sanitation, water, and hygiene (WASH) [12, 13], location [9, 14], contact with animals [15] and seasonal variations [16] are major risk factors in the transmission of parasitic infections, especially protozoan infections.

In Algeria, due to diversity in the socioeconomic status, and variation in geographic location, sanitary/hygiene and cultural factors of different communities, wide ranges of prevalence of intestinal parasites have been reported to-date (19.96% to 60.61%) in symptomatic and asymptomatic populations [17–19]. However, the implementation of appropriate control measures is dependent on precise local knowledge of the risk factors for intestinal parasitic infections and data on the prevalence of locally prevalent intestinal parasites is crucial in this context. For this, regional epidemiological studies are required throughout the country so as to enable optimization of appropriate control measures. No current information is available on the potential risk factors associated with intestinal protozoan infections in Algeria, particularly in the central Algerian province, Laghouat. This region is characterized by a diversity of human life styles and includes urban, rural and Bedouin communities. It is also known as the province with the second most numerous livestock ownership in Algeria, so this makes Laghouat an interesting area for parasitological research although to date there are no published reports on the extent of prevalence and distribution of human intestinal parasites among people living in this region. Therefore, the objective of the current study was to determine the prevalence of the most common intestinal parasites and to identify associated risk factors among symptomatic and asymptomatic populations in Laghouat province southern Algeria.

## Methods

### Study area

The study was carried out in the province of Laghouat, the province with the second most numerous livestock and one of the important socio-economical spots in Algeria, located in the center of the country at 400 km to the south of the capital Algiers (33° 48ʹ N, 02° 53ʹ E). The province of Laghouat is affected by three types of climates: semi-arid in the north, arid in the center and Saharan in the extreme south of the province. Daily temperatures average − 5 °C in winter months and over 40 °C in summer. The average annual rainfall is 151.21 mm per year [Unpublished observations, Bouchetata, 2018]. Laghouat province is located on the banks of the Mzee valley in the Amour Range of the Saharan Atlas mountains, and constitutes an oasis on the northern edge of the Sahara Desert. This province covers about 25,052 km^2^ and in 2016 had a population estimated at 603,876 inhabitant, of which 81.5% lived in an urban environment and 4.9% in rural areas of the province [Programming and Budget Monitoring Department, 2017]. These two populations live under essentially similar conditions with respect to local infrastructure and provision of drinking water, which is made available through boreholes and taps (supplied by wells or public water delivery systems). However, the rural population is mainly engaged in agricultural activities such as livestock husbandry and breeding (sheep, goats, cattle, horses, camels, chicken) [Directorate of Agricultural Services, 2016]. The remaining 13.6% of the population, the Bedouin (Nomads), live in sparsely populated parts of the province in relatively isolated communities, without infrastructure, with unsafe water supplies and inadequate levels of sanitation.

### Study population and sample collection

#### Study design

**Fig. 1:**
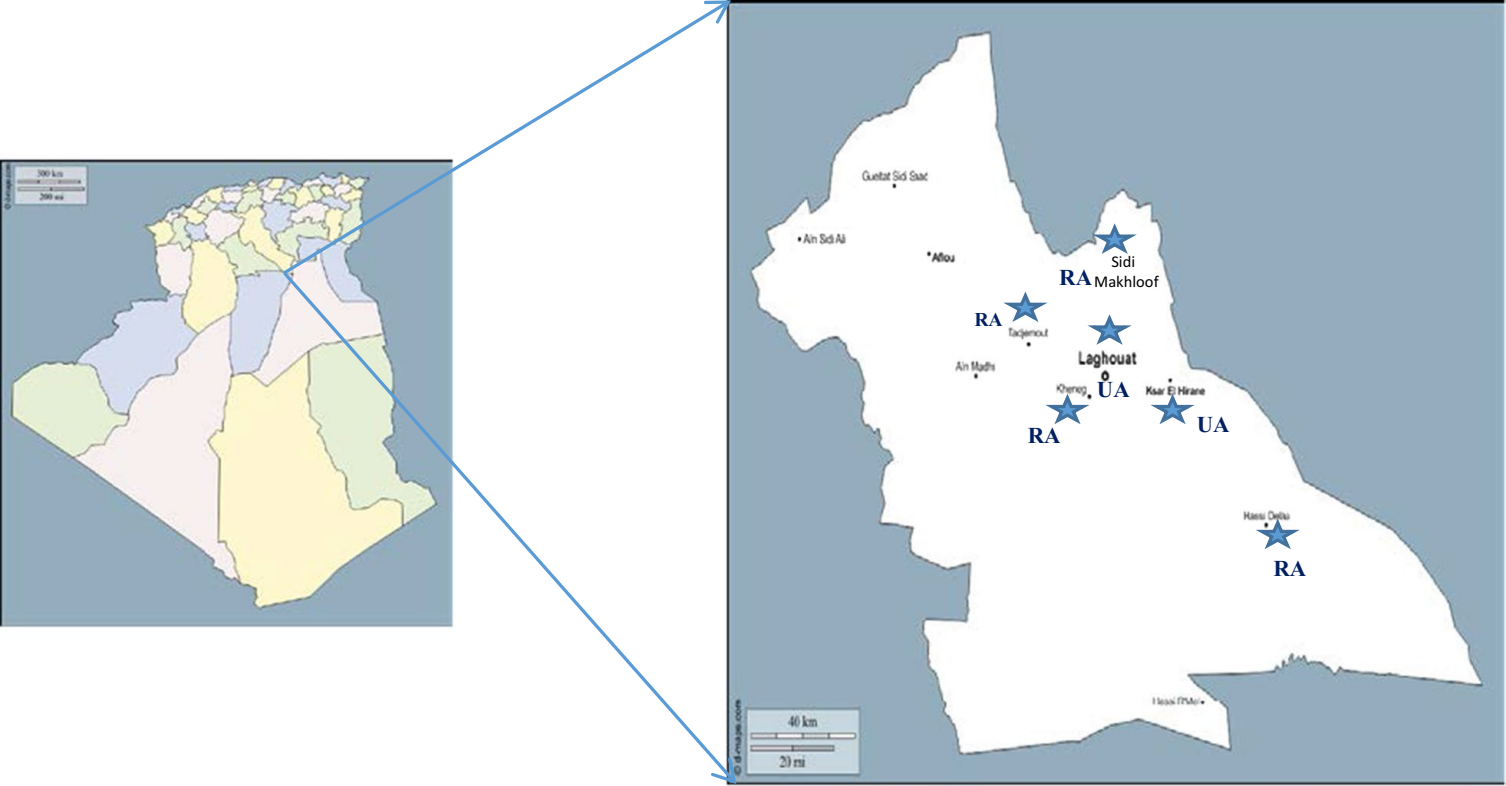
**A** Geographic map of Algeria, **B** Geographic map of Laghouat province showing the origin of the patients (*UA* urban areas, *RA* rural areas). Maps source: https://d-maps.com/carte.php?num_car=189308&lang=fr

A comparative cross-sectional study was carried out between, March 2015 and July 2018, among 2277, symptomatic and asymptomatic subjects aged 1–89 years (27.4 ± 18.66). Laboratory procedures were carried out in the central laboratory of the local public health hospital and in a private laboratory of medical analysis in Laghouat province, southern Algeria. The study populations were from two urban areas (Laghouat and Ksar El Hiran) and from four rural areas (Kheng, Sidi Makhlouf, Tadjmout and Hassi Delaa) (Fig. 1).

#### Data collection

Data for this study were collected from two subsets of the population; asymptomatic patients, designated hereafter as “Population 1”, were subjects referred to medical laboratories as part of regular medical examination or for provision of a medical certificate of health status required for job applications, for a VISA file processing and for patients with hypereosinophilia; symptomatic patients designated hereafter as “Population 2”, were subjects with gastrointestinal disorders who presented with one or more symptoms and were referred to medical laboratories for etiological diagnosis.

Structured questionnaire were prepared to gather relevant information from each subject. The objectives of this study were explained to the participants and a standard questionnaire summarizing personal information was completed by each individual or their guardian (i.e. age, sex, place of residence, water consumption, digestive disorders, immune status, and animal contact). The study was ethically approved by the Faculty of Science of Nature and Life, Djelfa University, Algeria (Ref:AT04/E.V.E.S) and informed consent was obtained from each patient or his/her parents or guardians in the case of minors.

### Sample processing

Fresh fecal specimens were collected from each subject in 25 ml clean wide-mouth, covered plastic containers and then immediately examined in normal saline (0.9%) and Lugol’s iodine preparations using direct microscopy, as part of a routine parasitological examination. After completing the direct stool examination, the formalin-ether concentration and Willis flotation techniques were performed in order to increase the likelihood of detection of parasites [20]. Additional individual fecal smears were fixed with methanol and stained by the modified Ziehl–Neelsen for detection of intestinal coccidian parasites. Stool samples suspected of containing *Blastocystis* were inoculated into 5 ml of modified Boeck and Drbohlav’s Locke-egg serum medium supplemented with 10% horse serum and incubated at 37 °C [21]. The cultures were checked for the presence of *Blastocystis*, by direct microscopy on the 3rd day after inoculation.

### Statistical analysis

Data was summarized as prevalence (percentage of infected subjects) ± with 95% confidence limits [CL_95_]) in the text and table, and as 95% confidence intervals [CI_95_]) in figures. Both CL_95_ and CI_95_ were calculated by bespoke software based on the tables of Rohlf and Sokal [22].

Analyses of data for combined protozoan spp. infection and for each of the protozoan infection measures (prevalence) were conducted using maximum likelihood techniques based on log linear analysis of contingency tables in the software package IBM SPSS (version 24). This approach is based on categorical values of the factors of interest, which are used to fit hierarchical log-linear models to multidimensional cross-tabulations using an iterative proportional-fitting algorithm and detect associations between the factors, in this case one of which is the presence/absence of infection (hereafter referred to as INFECTION, and referring to combined protozoan infection or specific parasites in each case at 2 levels, present or absent).

Analysis was carried out in two phases. First, exploratory models were fitted with the presence/absence of INFECTION relative to single explanatory factors of interest, in turn as described elsewhere [21]. For this phase we fitted in turn the following seven factors SEX, (at two levels, male or female), AGE (11 levels corresponding to the following age limits, level 1 = 1–4 years; level 2 = 5–9; level 3 = 10–14; level 4 = 15–19; level 5 = 20–24; level 6 = 25–29; level 7 = 30–34; level 8 = 35–39; level 9 = 40–44; level 10 = 45–54; level 11 =  > 54), AREA (location in which people lived either rural or urban), YEAR (4 levels, 2016–2018), SEASON (4 levels, spring, summer, autumn and winter), WATER (2 levels, subjects using bottled or tap water) and ANIMALS (subjects owned or did not own domestic animals).

In a second phase we fitted multi-factorial models as described previously [23], beginning with the full factorial model, and then employing the backward selection procedure in SPSS, we simplified the model until only significant terms remained. For each level of analysis in turn, beginning with the most complex model, involving all possible main effects and interactions, those combinations that did not contribute significantly to explaining variation in the data were eliminated in a stepwise fashion beginning with the highest level interaction (backward selection procedure). A minimum sufficient model was then obtained, for which the likelihood ratio of Chi-square was not significant, indicating that the model was sufficient in explaining the data. The importance of each term in interactions involving INFECTION in the final model was assessed by the probability that its exclusion would alter the model significantly and these values are given in the text, assessed by a likelihood ratio test between models with and without each term of interest.

Relationships between prevalence of infection and levels within specific factors that showed a directional trend (meaningful increase across levels, e.g. change with host age) were examined by the non-parametric Spearman’s test in SPSS 24, and *r*_*s*_ is given. *P* values less than 0.05 were considered to indicate statistical significance.

## Results

Of the 2277 subjects involving in the study, 759 (33.3%; CL_95_ 31.40–35.27) were infected with intestinal parasites, the majority of which were attributable to protozoan species (prevalence = 32.5% [30.62–34.47]), helminth infections in this population being relatively rare (prevalence = 0.79% [0.469–1.249]). However a significant difference was found in prevalence of parasitic infection between population 1 (14.9%; CL_95_ 13.16–16.59) and population 2 (82.3%; CL_95_ 79.55–84.84) and this difference in prevalence of combined protozoan infection between the two populations was highly significant (Fig. 3; *χ*^2^_1_ = 849.261, *P* < 0.0001). The overall prevalence of each of the species between the two populations is given in Table 1. The most common species found in population 1 was *Blastocystis* spp. (8%) followed by *E. coli* (3.3%), *E. histolytica/dispar* (2.5%) and more rarely *Endolimax nana*, *G. intestinalis* and *Trichomonas intestinalis*. No case of helminth infections was found in the asymptomatic population 1. Ten intestinal parasitic organisms were recorded from population 2. Among these the intestinal parasites, *Blastocystis* spp. (43.8%), *E. histolytica/dispar* (25.4%) and *G. intestinalis* (14.6%) were the most commonly found and the rarest was the nematode *T. trichiura*, which was recorded in only one subject. Because helminths were so rare in the study populations, further analysis is focused on infections by protozoan species, but it is of interest to note that of the 18 subjects infected with helminths, 16 were under 14 years in age, and 13 of these were attributable to *E. vermicularis*. Only two adult subjects were diagnosed with helminths, both in their 30 s and both with infections with *Tænia* spp.

**Table 1:** Prevalence of protozoan and helminth species by population

	Asymptomatic population (n = 1654)			Symptomatic population (n = 623)		
	n	Prevalence (%)	CL_95_	n	Prevalence (%)	CL_95_
*Blastocystis* spp.	132	8.0	6.67–9.29	273	43.8	40.46–47.23
*Entamoeba histolytica*/*dispar*	41	2.5	1.83–3.43	158	25.4	22.49–28.47
*Giardia intestinalis*	5	0.3	0.10–0.71	91	14.6	12.32–17.19
*Entamoeba coli*	55	3.3	2.45–4.26	39	6.3	4.75–8.13
*Endolimax nana*	28	1.7	1.12–2.45	12	1.9	1.18–3.13
*Cryptosporidium* spp.	0	0	0	6	1.0	0.49–1.90
*Trichomonas intestinalis*	3	0.2	0.04–0.53	20	3.2	2.19–4.67
*Enterobius vermicularis*	0	0	0	13	2.1	1.30–3.33
*Taenia* spp.	0	0	0	4	0.6	0.32–1.42
*Trichuris trichiura*	0	0	0	1	0.2	0.08–0.70

### Socio demographic and intrinsic factors affecting prevalence of combined protozoan parasites

**Fig. 2:**
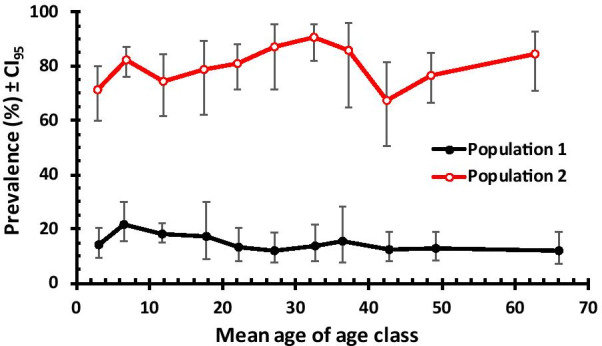
Prevalence of combined protozoan infection by host age class in POPULATION 1 and 2

The demographics and risk factors of the study subjects are shown in Tables 2 and 3. Prevalence of combined protozoan infection did not vary significantly between host age classes when tested separately within each population, (for population 1, *χ*^2^_10_ = 11.949, *P* = 0.289; for population 2, *χ*^2^_10_ = 16.668, *P* = 0.082). The age prevalence profiles for both populations are illustrated in Fig. 2, where the contrasting patterns of these profiles can be seen. For asymptomatic population, a correlational test of the mean age of age classes and prevalence, showed a significant negative relationship (*r*_*s*_ = − 0.727, *n* = 11, *P* = 0.011), prevalence falling with increasing host age. Prevalence, initially at 21.8%, increased by 8% from age class 1 to age class 2 and then fell as host age increased, to a low of 12% among the oldest age class (Fig. 2). While in symptomatic population there was no significant relationship between the mean age of age classes and prevalence (*r*_*s*_ = 0.227, *n* = 11, *P* = 0.5), there was some fluctuation in prevalence between age classes, with prevalence increasing from age class 9 to the oldest age class (Fig. 2).

**Table 2:** Factors associated with protozoan infections among the asymptomatic population 1 in Laghouat province

Factor	Level	*N*	Prevalence	CL_95_	OR	*z*	*P*
Sex	Male	985	14.6	11.76–17.96	1		
	Female	669	15.2	12.85–17.96	1.051	0.352	0.375
		(*χ*^*2*^_*1*_ = 0.12, *P* = 0.730)					
Area	Urban	949	12.5	9.94–15.6	1		
	Rural	705	18	15.33–21.02	1.533	**3.08**	**0.00**
		**(*χ***^***2***^_***1***_ = **9.48, *P* = 0.002)**					
Water	Bottled	827	13.1	10.60–15.97	1		
	Tap	827	16.7	13.91–19.86	1.333	**2.069**	**0.047**
		**(*χ***^***2***^_***1***_ = **4.31, *P* = 0.038)**					
Animal	No	1305	10.4	8.76–12.08	1		
	Yes	349	31.5	26.46–37.03	3.956	**9.383**	**< 0.0001**
		**(*χ***^***2***^_***1***_ = **83.65, *P* < 0.001)**					
Season	Spring	497	14.7	10.33–20.28	1.115	0.522	0.348
	Summer	368	16.3	12.36–21.08	1.262	1.067	0.226
	Autumn	475	14.9	10.68–20.43	1.138	0.617	0.33
	Winter	314	13.4	10.09–17.50	1		
			(*χ*^*2*^_*3*_ = 1.17, *P* = 0.76)				
Year	2015	345	13.3	9.91–17.67	1		
	2016	590	14.2	12.03–16.73	1.079	0.385	0.37
	2017	393	17.3	13.08–22.42	1.36	1.485	0.132
	2018	326	14.7	11.21–19.04	1.122	0.519	0.349
		(*χ*^*2*^_*3*_ = 2.62, *P* = 0.45)					

The statistical outputs that are significant are emphasized in bold

*OR* Odds ratio, *CL*_*95*_ 95% confidence interval

**Table 3:** Factors associated with protozoan infections among symptomatic population 2 in Laghouat province

Factor	Level	*N*	Prevalence	CL_95_	OR	*z*	*P*
Sex	Male	348	81	76.12–85.16	1	− 1.097	0.219
	Female	275	77.5	72.96–81.43	0.804		
		(*χ*^*2*^_*1*_ = 1.2, *P* = 0.273)					
Area	Urban	288	78.5	73.94–82.47	1		
	Rural	335	80.3	75.45–84.38	1.118	0.562	0.341
			(*χ*^*2*^_*1*_ = 0.316, *P* = 0.574)				
Water	Bottled	249	84.7	80.96–87.93	1		
	Tap	374	75.9	70.52–80.67	0.568	**2.644**	**0.012**
			**(*χ***^***2***^_***1***_ **=** **7.302, *P* = 0.007)**				
Animal	No	327	73.4	68.24–78.00	1		
	Yes	296	86.1	82.12–89.43	2.255	**3.877**	**0.0002**
			**(*χ***^***2***^_***1***_ **=** **15.818, *P* < 0.001)**				
Season	Spring	182	86.3	78.51–91.67	3.581	**4.852**	**< 0.0001**
	Summer	85	90.6	80.34–95.97	5.489	**4.247**	**< 0.0001**
	Autumn	166	84.3	76.85–89.91	3.071	**4.291**	**< 0.0001**
	Winter	190	63.7	54.00–72.51	1		
			**(*χ***^***2***^_***3***_ **=** **41.046, *P*** **=** **0.001)**				
Year	2015	91	86.8	75.21–93.60	1		
	2016	199	88.4	80.59–93.56	1.162	0.395	0.369
	2017	151	83.4	76.15–88.94	0.766	− 0.704	0.311
	2018	182	62.6	53.19–71.25	0.255	− **3.971**	**< 0.001**
			**(*χ***^***2***^_***3***_ **=** **43.271, *P* < 0.001)**				

The statistical outputs that are significant are emphasized in bold

*OR* Odds ratio, *CL*_*95*_ 95% confidence interval

Prevalence values for male and female subjects from population 1 (*χ*^*2*^_*1*_ =0.12, *P* = 0.730) and population 2 (*χ*^*2*^_*1*_ = 1.2, *P* = 0.273) are summarized in Tables 2 and 3. Prevalence was similar in both sexes and there was no overall significant difference in either population.

**Fig. 3:**
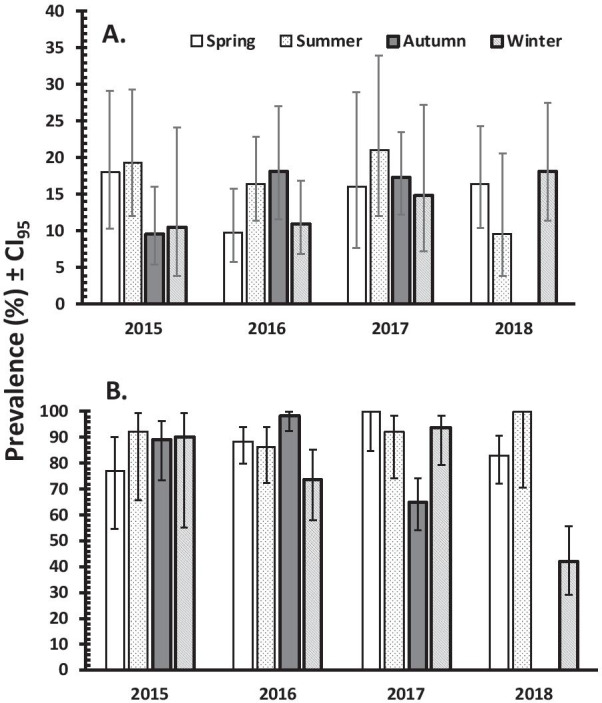
Prevalence of combined protozoan infection by year and season in POPULATION 1 (**A**) and 2 (**B**). Note that no data were collected in the autumn of 2018

For asymptomatic population, univariate analyses showed significant effects between combined protozoan infection and area in which subjects lived (higher in rural areas), the type of water utilised by subjects (higher for tap water) and animal ownership (higher among those who owned animals) (Table 2). Overall prevalence was similar in each of the years of the study varying only between 13.3% (2015) and 17.3% (2017). Prevalence was even more constant when examined by season, varying only from 13.4 to 16.3%. Neither of these was significant (Fig. 3A).

For symptomatic population significant factors included water (higher among those utilizing bottled water), ownership of animals (higher among those with animals), season (highest in summer and lowest in winter) and year (highest in 2016 and lowest in 2018) were recorded (Fig. 3B). Although there was no significant difference in prevalence between areas in which people lived (Table 3).

### Potential risk factors associated with combined protozoan infection

For population 1, multifactorial log-linear analysis showed that, with all relevant significant factors taken into account from the first round of analysis, prevalence of combined protozoan infection was significantly affected only by two factors. Ownership of animals retained significance (*χ*^*2*^_*1*_ = 83.6, *P* < 0.001), as did also the area in which people lived (*χ*^*2*^_*1*_ = 9.480, *P* = 0.002). With these two factors taken into account, water which showed only marginal significance in the exploratory analyses was no longer significant (*χ*^*2*^_*1*_ = 2.046, *P* = 0.153).

Multifactorial analysis of prevalence in population 2 confirmed that with other factors taken into account, ownership of animals retained significance (*χ*^*2*^_*1*_ = 4.831, *P* = 0.028). Utilization of different types of water lost significance (*χ*^*2*^_*1*_ = 0.969, *P* = 0.325) but there was a highly significant interaction between season and year (SEASON × YEAR × INFECTION, (*χ*^*2*^_*9*_ = 54.652, *P* < 0.001). This is illustrated in Fig. 3B, which shows that prevalence was lower in the autumn of 2017 and winter of 2018 than among the other seasons of the 4 year period.

### Risk factors associated with *Blastocystis* spp. and *G. intestinalis* infection

**Fig. 4:**
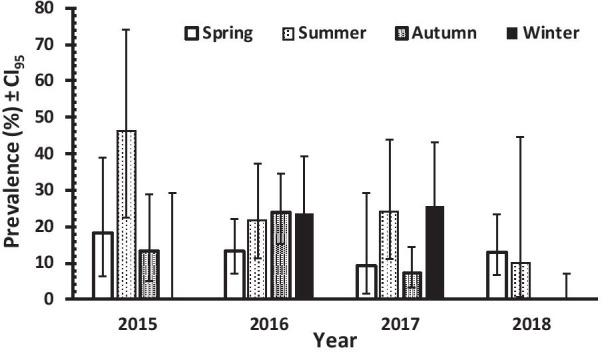
Prevalence of *G. intestinalis* infections by year and season in POPULATION 2

We explored next the effects of the explanatory factors on the two most prevalent species in the symptomatic population. For *G. intestinalis,* the minimum sufficient model retained two interactions with INFECTION. The first was that between YEAR, SEASON and INFECTION (*χ*^2^_9_ = 33.416, *P* = 0.001), and this is illustrated in Fig. 4. Prevalence of *G. intestinalis* fluctuated significantly between years and seasons of the study. Nevertheless high prevalence was detected in summer 2015, whereas in winter 2015 and 2018 no cases were detected. In 2016, prevalence was similar between winter (23.7%), autumn (23.8%) and summer (21.6%). In 2017 prevalence was high in summer (24%) and winter (25.5%) and low in spring (9.1%) and autumn (7%). Low prevalence was recorded in the spring and summer of 2018, but note that no data were collected in the autumn of 2018. Overall, however, when prevalence in each season of the 4 years is considered, prevalence of giardiasis appears to be higher in the dry seasons of summer and spring.

**Fig. 5:**
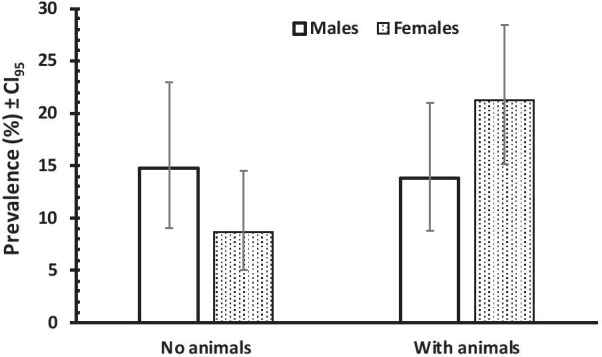
Prevalence of *G. intestinalis* in male and female subjects with or without animals in POPULATION 2

The second significant interaction was that between SEX, ANIMALS and INFECTION with *G. intestinalis* (*χ*^2^_1_ = 5.543, *P* = 0.019). Prevalence was higher in female subjects (21.2%) that owned animals, than in male subject (13.8%). In contrast prevalence in male subject, with no contact with animals, was higher than among female subject with prevalence of 14.8% and 8.7%, respectively (Fig. 5).

**Fig. 6:**
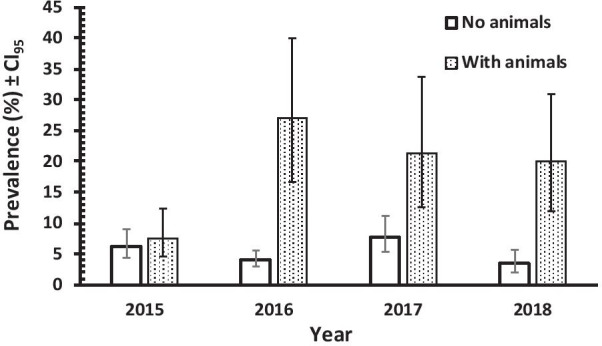
Prevalence of *Blastocystis* spp. in subjects with or without animals during the 4 years of the study, in POPULATION 1

Multifactorial analysis of *Blastocystis* infection resulted in seven different interactions with INFECTION, some comprising 3 and 4 factors each and these were not explored further. Of interest was the interaction in population 1 between ANIMAL × YEAR × INFECTION (*χ*^2^_1_ = 27.463, *P* = 0.001) and this is illustrated in Fig. 6, where it is clearly apparent that the possession of animals was a key determinant of prevalence. This was consistently higher among those owning animals in each year of the study, although in 2015 the difference between those that had animals and those that did not, was very small.

Multifactorial analysis of prevalence of *Blastocystis* in population 2 yielded two expressions of interest in the minimum sufficient model. The first comprised four factors along with INFECTION, and this was not considered further. The second expression showed that with all the other factors taken into consideration, prevalence differed significantly between the sexes (*χ*^2^_1_ = 7.342, *P* = 0.007) in population 2. Prevalence was significantly higher among male subjects (48.6%; CL_95_ 42.88–54.25) compared with females (37.8%; CL_95_ 33.03–42.86).

## Discussion

In this study we carried out a comparative analysis of intestinal parasitosis between symptomatic subjects who suffer from gastrointestinal disorders (82.3%) and asymptomatic subjects representing the background population (14.9%). Risk factors according to the demographic, spatio-temporal and behavioral were evaluated. A couple of previous studies from North Algeria have reported on prevalence of intestinal parasitic infection among symptomatic and asymptomatic (combined) patients, 27.6% in Algiers [18] and 60.61% in Blida [19]. Similar studies have been conducted also in neighbouring countries, 26.6% in Tunisia [24] and 14.15% in Morocco [25]. Our current study, is the first investigation of the risk factors affecting the spread of intestinal parasites in Algeria.

Variation in overall prevalence of intestinal parasitic infections among North African countries may be attributable to variation in the sampling techniques used, dissimilarity in the selection of the enrolled study population, differences in WASH quality, close contact with animals and variation in the environmental conditions in the different study locations. In our study, no case of helminth infection was found in the asymptomatic population while in the symptomatic population, a low prevalence (2.9%) was detected, represented by *E. vermicularis*, *Tænia* spp. and one case of *T. trichiura*. This finding is in agreement with earlier studies conducted in Algeria [17–19], in North Africa [9, 24, 25] and in Europe [26, 27] but differs from studies carried out further south in Africa and elsewhere in the tropics, in which soil-transmitted helminthiasis have been reported to be the dominant parasites [14, 28].

In this study, several possible determinants associated with intestinal parasitic infections were investigated. When all the factors were fitted in a multifactorial model, the most marked and most consistent influence on prevalence of combined protozoa infection in both populations was ownership of animals. Subjects who owned or looked after animals had a markedly higher prevalence of infection. The next influence on prevalence in terms of the magnitude of its effect in asymptomatic population was the location in which subjects lived. Overall data showed that those living in rural locations had higher prevalence of protozoan infections, but the magnitude of this effect was not nearly as marked as that of ownership of animals. This may be explained by the precarious hygienic conditions in rural areas where access to adequate sanitation and the supply of drinking water is difficult. Improved access to WASH may prevent an estimated 8% of deaths and 10% of the disease burden in developing countries) [29]. The other relevant factor in Laghouat rural area is close contact with livestock. Additionally, nomadic and pastoral populations live close to their animals and this close contact increases the risk of zoonotic transmission. Livestock, have often been implicated as a major source of environmental contamination and potential reservoirs for zoonotic infection [30–32]. In Algeria *G. intestinalis* and *C. parvum* have been reported in domesticated animal species [30–32]. These parasites are potentially zoonotic pathogens and close contact with animals is believed to be the major risk factor for human infections.

In symptomatic population, the prevalence of combined protozoan infection was influenced by season and year of assessment, with the highest prevalence of infection recorded in summer and in 2016. These fluctuations in prevalence are most likely explained by seasonal changes in eating habits in particular years, with an increase in the consumption of water and raw foods during the hot seasons, communal swimming and on variation in climatic conditions which favour the embryonation and development of the infective stages in ova and oocysts during their external phase. Another pertinent factor centres around agricultural practices in the spring in the Algerian steppe. This is the best time for pasture improvement and in this period livestock owners and breeders are in close contact with their livestock, because this is the season when goat kids and lambs are born and when sheep are sheared. Transhumance of animals in this period is very frequent in search of fresh pastures on which to graze and this close contact between livestock and pastoralists increases the risk of zoonotic transmission. This result is consistent with our finding that ownership of animals in Laghouat province is by far the most important risk factor predisposing people to infection with protozoan parasites in both populations.

In addition, the overall pattern of the prevalence of combined protozoan infection in symptomatic population was clearly influenced by the pattern of prevalence of *Blastocystis* spp., *E. histolytica/dispar* and *G. intestinalis,* the dominant species*.* The high prevalence of *Blastocystis* spp. in symptomatic population confirms the results of a previous survey in Libya in which a prevalence of 35.3% was recorded in symptomatic patients compared to a prevalence of 13.2% in asymptomatic subjects [33]. However, multifactorial analysis of prevalence of *Blastocystis* infection and risk factors showed that in symptomatic population male subjects experienced significantly higher prevalence with *Blastocystis* than females. The higher prevalence in male subjects might be due to their greater everyday participation in outdoor activities than females, as for example in the raising and husbandry of animals, which make them more vulnerable to parasitic infections. Moreover, in asymptomatic population, significantly higher values for prevalence of *Blastocystis* infection were recorded in each year of the study among those who owned animals. It is pertinent that a high prevalence of *Blastocystis* infections, and subtypes of this species, have been previously reported amongst subject with close contact with animals and animal handlers [34, 35], providing supporting evidence that transmission of the parasite between humans and animals may be frequent in pastoral communities.

The prevalence of *E. histolytica/dispar* was significantly higher among symptomatic patients, 10 time higher than observed in asymptomatic ones. In this study we did not distinguish between the pathogenic *E. histolytica* and the non-pathogenic *E. dispar*, although the non-pathogenicity of latter species has been questioned. An earlier study has suggested the existence of several different genotypes of *E. dispar* that can be associated with, or be potentiality responsible for, intestinal or liver tissue damage, similar to that observed with *E. histolytica* [36]. Moreover, other studies have identified also *E. dispar* in symptomatic patients [37, 38]. So the high rate of infection in symptomatic patients observed in the current study may be due to the presence of either or both *E. histolytica* and *E. dispar*. This should be confirmed by further studies, incorporating molecular diagnostic tools to distinguish between these different species of *Entamoeba* and their variants.

In our study the prevalence of *G. intestinalis* was significantly higher in symptomatic patients, similar finding having been found previously in west and north Algeria [17, 19]. In our study we recorded a high prevalence of *G. intestinalis* in spring and summer in symptomatic population, a result that is consistent with earlier studies that have observed a seasonal pattern of giardiasis peaking in mid-summer and late winter, as for example in a study of Egyptian children [39] and another reporting high prevalence in summer and fall in Iran [40]. Several factors could account for seasonal variation in the occurrence of giardiasis, including eating habits, agricultural practices, humidity or temperature and factors promoting exposure to cysts, such as contact with animals. Indeed, we found that prevalence was significantly higher in female subjects who had contact with animals compared with male subjects in symptomatic population. This may be due to the fact that females often have more intimate relationships with their pets than males. A study in Mexico showed that pets and their owners share the same subgenotype of *G. intestinalis* with a higher prevalence in female than male subjects [41]. The predominance of these parasites in symptomatic patients is an indicator of the pathogenicity of these parasites and reflects the poor hygiene practice of this population.

Our data showed that prevalence of combined protozoan infection did not vary significantly between the sexes in either population. Nevertheless we observed an interesting interaction between the sexes, ownership of animals and infection with *G. intestinalis,* and between the sexes and infection with *Blastocystis* spp. Also prevalence showed no significant variation with host age in either population, however, prevalence was high in school children age with significant negative trend in asymptomatic subjects, but no such relationship was evident for the symptomatic patients, much in line with studies elsewhere [9, 24, 29]. The relatively higher prevalence in school children could be explained by the fact that children in this age are more susceptible to intestinal infectious diseases than adults because of their poor hygiene habits, poor health education, consumption of school tap water, immature immunity of young children and close personal contact in crowded classrooms [29].

Our study has shown that contact with animals was the most important risk factor and thus predictor of intestinal protozoan parasitic infections in both populations in Laghouat province. This analysis also revealed that living in rural, rather than in urban areas, whilst showing less temporal stability, was nevertheless a significant additional secondary risk factor that also needs to be taken into consideration. Further molecular investigations of the exact genotypes of each of the intestinal parasites recorded here in humans, and extended additionally to animals and tap water samples, are required to confirm these findings.

## Conclusion

To the best of our knowledge, this is the first study reporting epidemiological data on intestinal protozoa infection among symptomatic and asymptomatic patients in Algeria. The findings of this study emphasize that intestinal parasitic infections is a major public health challenge in Algeria and provide crucial information about the risk factors for both populations, and on this basis appropriate strategies for control, aiming to effectively reduce the prevalence of these infections, should focus primarily on improving animal husbandry practices, but also additionally on health education to improve personal hygiene practices, and on providing safe drinking water and adequate sanitation.

## Data Availability

The datasets used and/or analyzed during the current study are available from the corresponding author on reasonable request.

## Abbreviations

PSR: Protozoan species richness
S.E.M: Standard error of the mean
GLM: Generalized linear model
CL: Confidence limits
OR: Odds ratio
WASH: Water, sanitation and hygiene
